# Microfluidic Assay Measures Increased Neutrophil Extracellular Traps Circulating in Blood after Burn Injuries

**DOI:** 10.1038/s41598-018-34952-0

**Published:** 2018-11-19

**Authors:** Masayuki Otawara, Maedeh Roushan, Xiao Wang, Felix Ellett, Yong-Ming Yu, Daniel Irimia

**Affiliations:** 1 BioMEMS Resource Center, Division of Surgery, Innovation and Bioengineering, Department of Surgery, Massachusetts General Hospital, Boston, Massachusetts United States of America; 2 Harvard Medical School, Boston, Massachusetts United States of America; 3 Shriners Burns Hospital, Boston Massachusetts, United States of America; 4 Kyorin University, School of Medicine, Department of Traumatology and Critical Care Medicine, Tokyo, Japan

## Abstract

Cell-free DNA (cf-DNA) concentration in human plasma is often increased after burn and trauma injuries. Two major sources of cf-DNA are the parenchymal cells damaged by the injury and various circulating cells indirectly altered by the response to injury. The cf-DNA originating from neutrophils, also known as circulating neutrophil extracellular traps (cNETs), is of notable interest because cNETs have been associated with pathological processes in other conditions, including cancer, autoimmunity, etc. Both intact chromatin and oligonucleotides, which are the by-product of cf-DNA degradation, are assumed to contribute to the cf-DNA in patients. However, traditional assays for cf-DNA quantification do not distinguish between cNETs and cf-DNA of other origins and do not differentiate between intact chromatin and oligonucleotides. Here we measure the amount of intact cNETs in the circulation, using a microfluidic device that mechanically traps chromatin fibers directly from blood and an immunofluorescence protocol that detects neutrophil-specific proteins associated with chromatin. In a rat model of burn injury, we determined that the chromatin fibers in the circulation after injury originate exclusively from neutrophils and are cNETs. We found that the concentration of cNETs surges the first day after injury and then decreases slowly over several days. In a secondary sepsis model, which involved a burn injury followed by cecal-ligation-puncture, we measured additional increases in cNETs in the days after sepsis was induced. These results validate a microfluidic assay for the quantification of cNETs and will facilitate fruther studies probing the contribution of cNETs to complications after burns and sepsis.

## Introduction

Neutrophils are innate immune cells that defend against pathogen invasion. To immobilize and damage microbes, neutrophils can undergo a specialized process by which they release their nuclear chromatin and antimicrobial granule proteins, which together form Neutrophil Extracellular Traps (NETs)^1^. NETs released in tissues have been shown to contribute to protection against microbes^1^. However, NETs can also contribute to organ damage^2,3^. When NETs are released in the circulation (circulating NETs - cNETs), they have also been linked to complications in various diseases^4^.

Severe burn injuries are often complicated by organ failure and are associated with high mortality rates^5,6^. Circulating cell-free DNA (cf-DNA) is increased after burn injury, proportional to the severity of the burns and the length of hospital stay after burn injuries^7–9^. Recently, correlations have been reported between cf-DNA and complications after sepsis^10^, trauma^11^, cancer^12^, pregnancy^13^, and cardiovascular disease^14^. However, how cf-DNA contributes to pathology after burn injuries^15,16^ and the cellular origin of cf-DNA remains controversial^9^. A handful of studies suggested that the cf-DNA originates from both neutrophils (cNETs) and damaged cells following burn injury and trauma^17^.

Several studies have proposed an association between cNETs trapped in capillary vessels and subsequent organ dysfunction^18,19^. A biochemical mechanism is commonly assumed to be involved, centered on evidence of cNETs-induced thrombosis *in vitro*^20^. A mechanical mechanism has also been proposed, when the trapping of cNETs inside *in vitro* capillary plexuses perturbs the distribution of flowing red blood cells, and may be responsible for localized tissue hypoxia and damage^21^. For this mechanism, the distinction between circulating chromatin fibers and oligonucleotides is critically important. Oligonucleotides pass easily through capillaries, while chromatin fibers could become entangled inside the capillary plexuses^21^. Chromatin fibers also may produce larger thrombi than oligonucleotides^20^. DNA degrading enzymes, such as DNase1, are present in plasma^20^, and could possibly degrade cNETs to oligonucleotides. However, no current assay for NETs could distinguish between the intact and degraded chromatin fibers based on differences in size. Traditional cf-DNA biochemical assays are sensitive to both the chromatin and oligonucleotides and thus cannot distinguish between the two^22,23^.

Here, we validate a microfluidic assay for measuring intact cNETs in blood. We apply the new tool to analyzing blood from rats after burn injury and sepsis. Using our new microfluidic technique and immunostaining for neutrophilic elastase (NE) we show that the captured chromatin from blood is cNETs. Moreover, we quantify the amount of cNETs and show that cNETs increase rapidly after burns and decrease slowly in the days after the injury. Secondary sepsis induced by cecal ligation puncture at day 9 post burn results in additional, progressive increase in the concentration of cNETs for several days.

## Results

### The microfluidic device for capturing chromatin from blood

**Figure 1:**
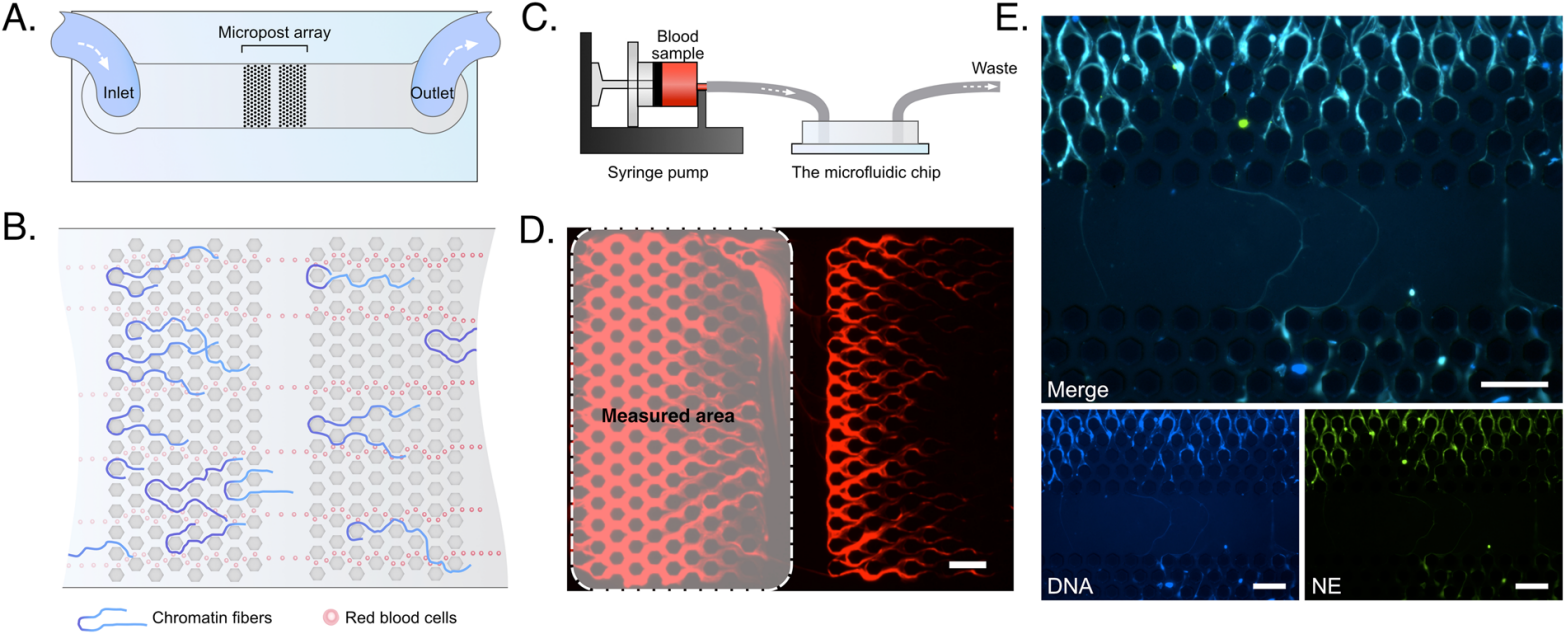
Microfluidic device for measuring circulating chromatin. (**A**) A schematic showing the geometry of the microfluidic device. (**B**) A schematic illustrating the trapping of chromatin fibers by the post-array in the device. (**C**) A schematic showing the operation system for the microfluidic device. A blood sample is loaded in a 1 mL syringe and introduced into the microfluidic device at 10 µL/min with a syringe pump. (**D**) A fluorescent microscopic image showing the chromatin fibers trapped in the post array. The dashed rectangular region indicates the measurement location. The chromatin was stained with Sytox-orange. Scale bar is 100 µm. (**E**) Immunofluorescence staining of cNETs in the microfluidic device. cNETs are trapped inside the microfluidic device from blood collected at 1 PBD after 30% TBSA burn. cNETs are stained with Sytox-blue (blue). Neutrophil elastase (NE) is identified by immunofluorescence staining (green) and is co-localized with the chromatin fibers trapped on chip, validating their neutrophil origin. Individual images for each fluorescence channel and a merged image are presented. Scale bars are 100 µm.

We designed a microfluidic device to capture chromatin from blood. The device consists of two arrays of micro-posts arranged in series in a straight microchannel (Fig. 1A). The distance between the posts is 20 µm. The height of the device is 35 µm. The distance between adjacent posts is optimized to trap de-condensed chromatin fibers efficiently yet let blood cells and oligonucleotides flow through (Fig. 1B). To capture chromatin from blood, whole blood samples are first diluted 80 times. The samples are loaded to 1 mL syringes, mounted on syringe pumps, and pumped through the device with a flow rate of 10 µL/min (Fig. 1C). As the samples flow through devices, chromatin fibers are captured in the post array (Fig. 1D). We quantified the amount of chromatin by measuring the area of the fluorescent region in the first array and employed the second array to determine when the chromatin capture in the first array reaches saturation.

### Circulating chromatin after major burns is derived from neutrophils

We probed the origin of the chromatin in blood after major burns using immunofluorescence staining for neutrophil elastase (NE). We found that NE is associated with the chromatin trapped in the devices. This result suggests that the circulating chromatin in the blood of rats after major burn is predominantly derived from neutrophils (cNETs - Fig. 1E).

### The microfluidic assay measures intact chromatin

**Figure 2:**
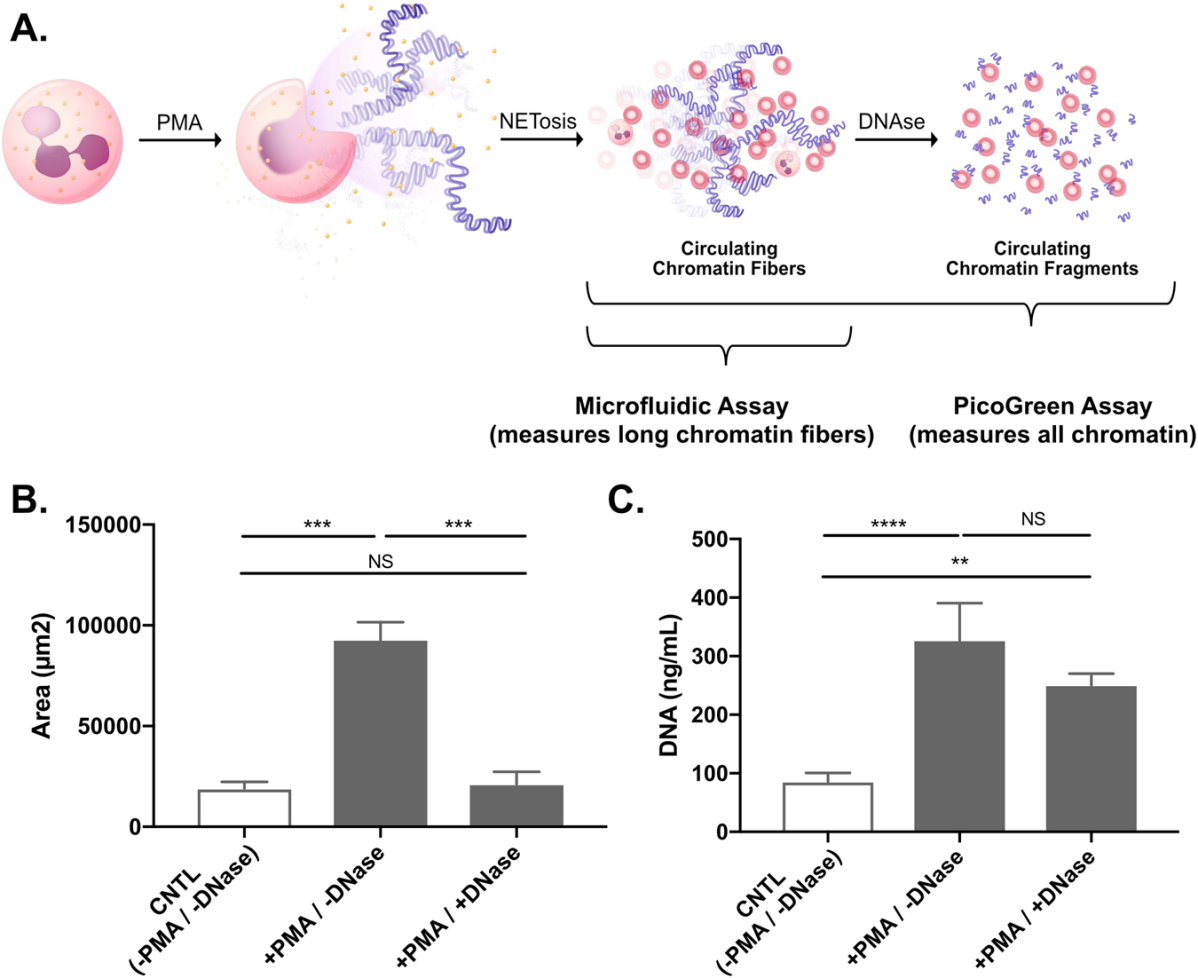
Quantification of cNETs in blood using microfluidics and PicoGreen assay. (**A**) Neutrophils release chromatin (NETs) following stimulation with PMA. The chromatin is then degraded by DNases into oligonucleotides. (**B**) The area of trapped chromatin in microfluidic devices significantly increases after PMA stimulation (+PMA/−DNase group). The area significantly decrease with the addition of DNase). There are no significant differences between the untreated control and the +PMA/+DNase group. (**C**) Plasma genomic DNA values measured using the PicoGreen assay are significantly increased both in +PMA/−DNase and +PMA/+DNase groups compared to controls. The differences between the +PMA groups with and without DNase are not significant. (N = 16 rats total, n = 1 experiment for each group. Data were compared using Friedman test; **p ≤ 0.01, ***p ≤ 0.001, ****p ≤ 0.0001).

We compared the measurements of chromatin in blood using the microfluidic assay with those using a PicoGreen-based assay. We used blood from healthy rats and stimulated the release of chromatin from neutrophils using phorbol 12-myristate 13-acetate (PMA). Both the microfluidic and PicoGreen assay measured significant increases in the amounts of chromatin in blood compared to control samples. When the stimulation of chromatin release was accompanied by degradation using DNase, the chromatin levels measured using the microfluidic assay were significantly reduced. In contrast, measurement using traditional PicoGreen assay did not change significantly (Fig. 2A,B). The results of these experiments show the microfluidic assay is specific for intact chromatin, while the traditional PicoGreen assay does not distinguish between the intact chromatin and the oligonucleotides resulting from its degradation.

### Neutrophils are the only source of cNETs after burn injury

**Figure 3:**
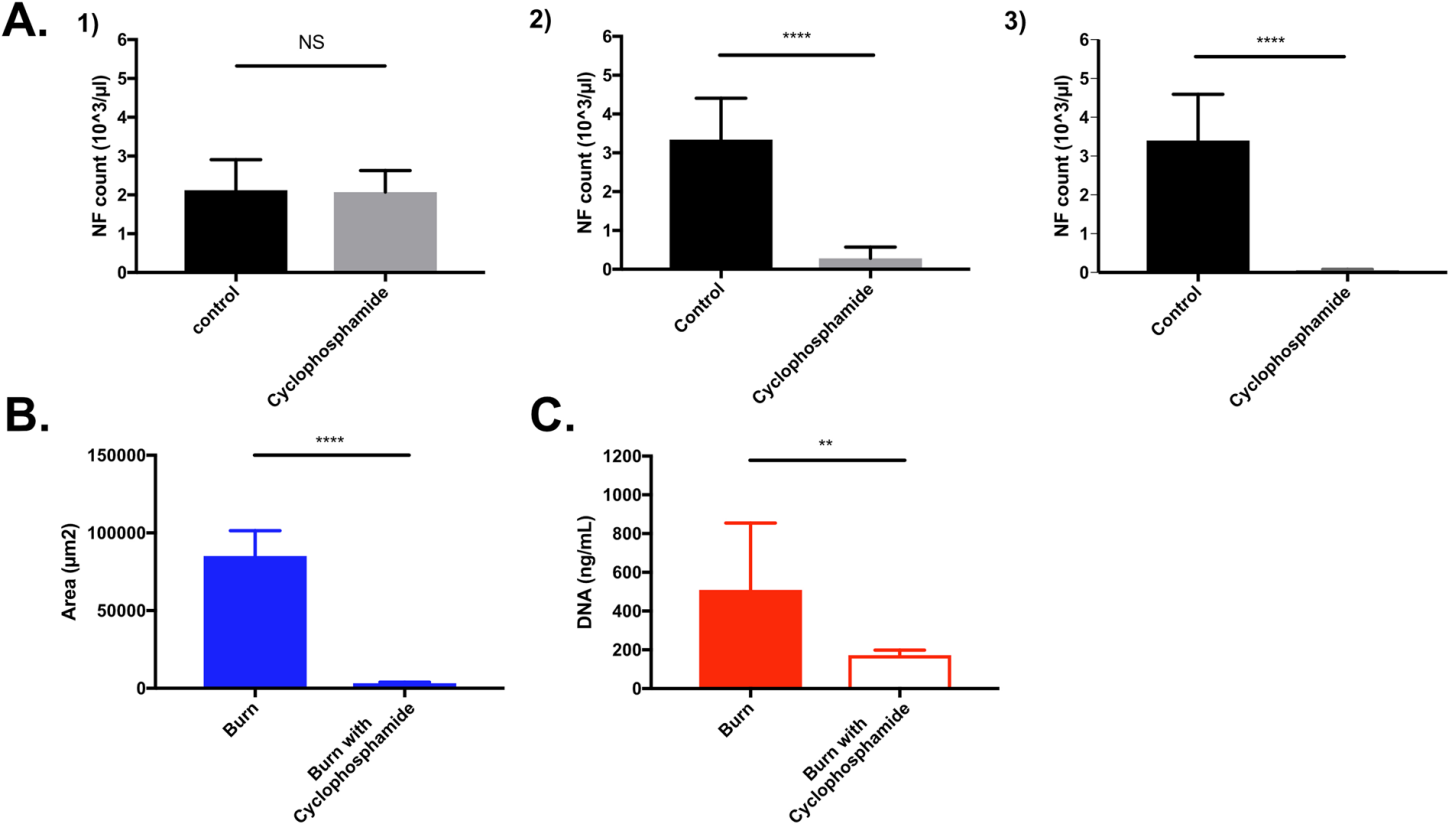
Neutrophil counts and cNETs amounts are measured in a rat model of neutropenia and burn injury. (**A**) Neutrophil counts (NF count) were performed at Day -4 i.e. before cyclophosphamide IP injection (#1), Day 0 before burn (#2), and one day after 30% TBSA burn injury (#3). Neutrophil counts significantly decreased in the cyclophosphamide group at Day 0 and 1 PBD. (N = 8 rats. Data were compared using Mann-Whitney test; **p ≤ 0.01, ****p ≤ 0.0001). (**B**) Area of cNETs trapped in microfluidic device after 30% TBSA injury is significantly lower in neutropenic rats compared to controls (N = 11 rats). (**C**) Plasma DNA values measured using PicoGreen assay are also significantly decreased in neutropenia rats after 30% TBSA injury compared to controls after 30% TBSA injury (N = 5 rats). The values form cyclophosphamide-treated and control rats are reported at 1 PBD. (Data were compared using Mann-Whitney test; **p ≤ 0.01, ****p ≤ 0.0001).

We measured the amount of cNETs after major burns in neutropenic rats. First, we verified that all the rats had comparable granulocyte counts before the administration of cyclophosphamide (pre-burn day 4 - Fig. 3A). Then, we verified that the number of neutrophils in the circulation was decreased after the administration of cyclophosphamide, just before the burn injury (pre-burn day 0). The number of neutrophils remained low at 1 post burn day (1 PBD). Measurements of circulating chromatin at 1 PBD showed significant differences between the control and cyclophosphamide groups. In the cyclophosphamide-induced neutropenia group, the area of chromatin trapped in the microfluidic device and total plasma genomic DNA value measured with PicoGreen cf-DNA assay were significantly lower than in the control group (Fig. 3B,C). These results suggested that the circulating chromatin after major burns is derived from neutrophils (cNETs). They also suggest that the contribution of the chromatin released from burn-damaged tissue to the measured cNET values is insignificant. Additional measurements in the blood samples from control and cyclophosphamide-treated rats at 1 PBD revealed that the platelet count also decreased significantly (from 600 ± 150 vs. 100 ± 25 × 10^3^/µL) while hemoglobin levels did not change (10.8 ± 3.7 vs. 10.6 ± 3.5 g/dL). Together, the results from neutropenic rats and the NE immunostaining indicate that neutrophils are the major source of cNETs after burn injury.

### Transient increases in cNETs after major burns

**Figure 4:**
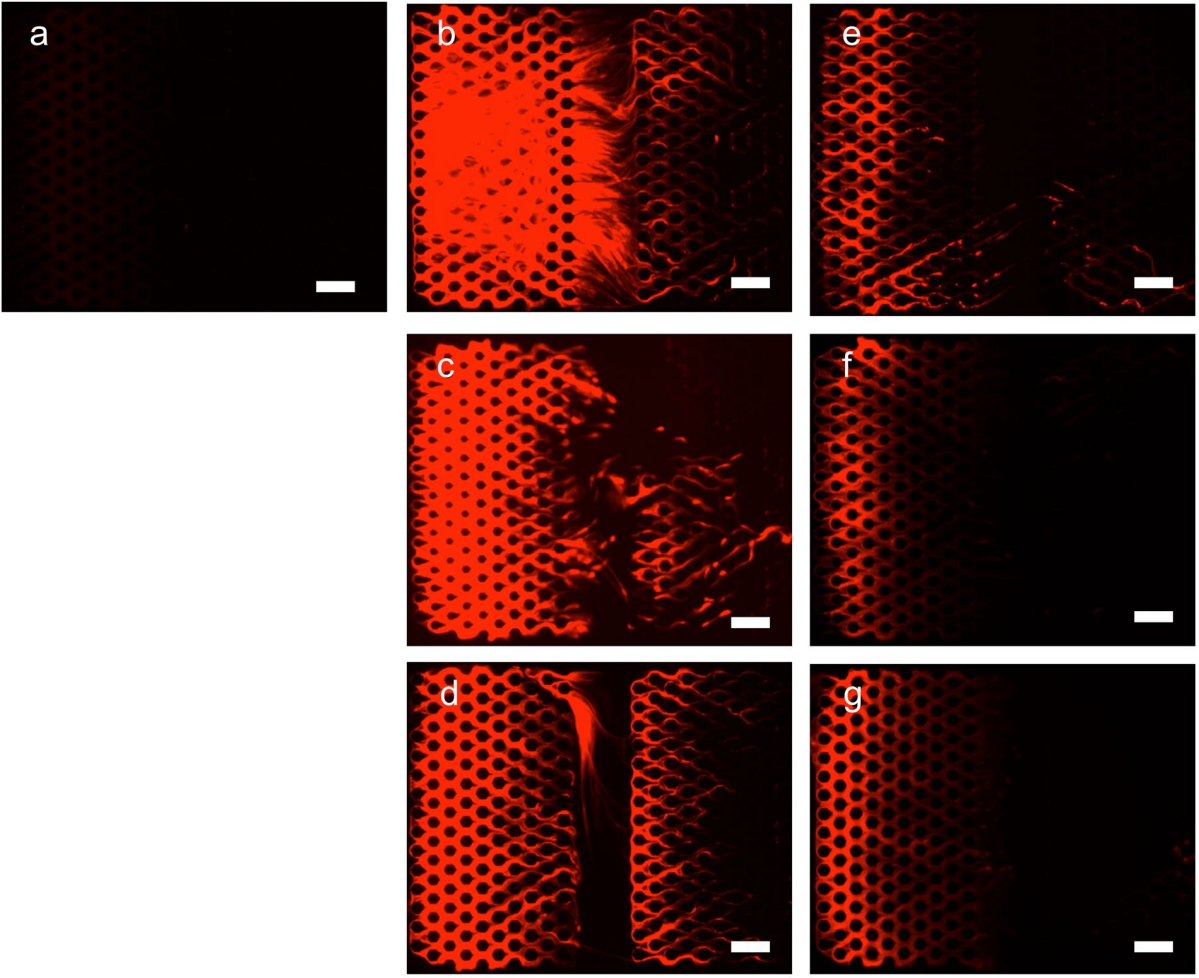
Representative images of chromatin trapped inside microfluidic devices from blood samples from rats after burn injury. Chromatin is stained inside the devices. Images are recorded: (**a**) before burn, (**b**) at 1 PBD, (**c**) 3 PBD, (**d**) 7 PBD, (**e**) 9 PBD and 6 hours after CLP, (**f**). 10 PBD, and (**g**) 11 PBD. Scale bars are 100 µm.

**Figure 5:**
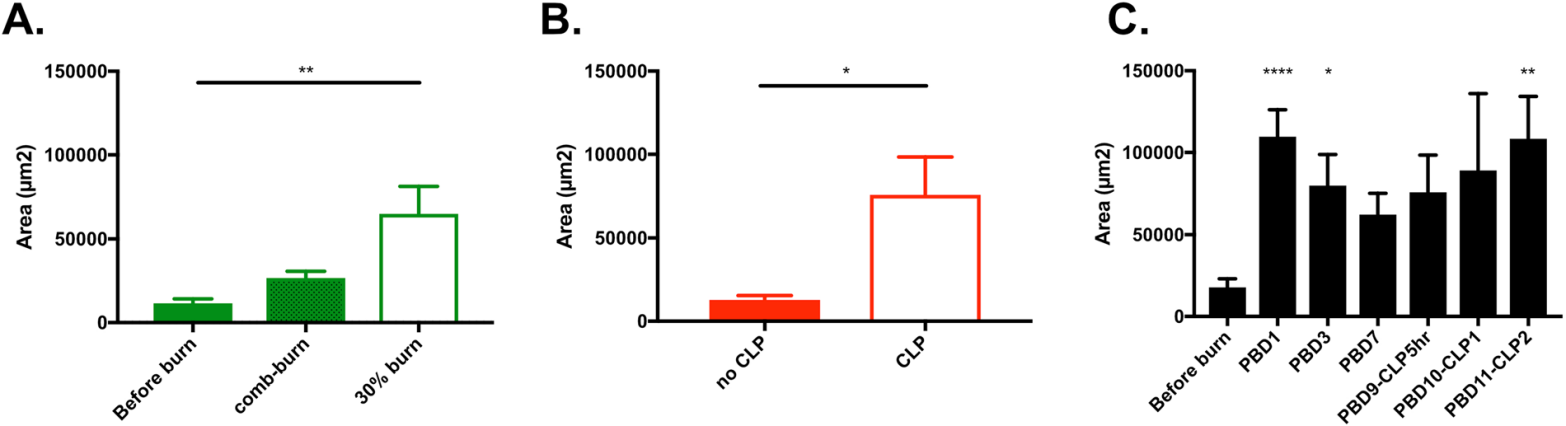
cNETs levels increase after burn and sepsis. (**A**) Significantly more cNETs are trapped in the microfluidic device after small (comb burn) and large (30% TBSA) burns in rats at 1 PBD (N = 8–13 rats for each group). Data represents the area of trapped cNETs in the devices. Comparisons are performed using the Kruskal-Wallis test; **p ≤ 0.01. (**B**) Significantly more cNETs are trapped in the microfluidic device from blood of 30% TBSA rats, at 9 PBD and 6 hours after CLP compared to the no CLP (N = 5–8 rats for each group). Data were compared using Mann-Whitney test; *p ≤ 0.05. (**C**) The area of trapped cNETs in microfluidic devices surges at 1 PBD (N = 8–18 rats for each group). The trapped cNETs area decreases slowly in the days after the injury. The trapped cNETs area increases steadily during sepsis after cecal ligation puncture. Data at each timepoint were compared using Kruskal-Wallis test; *p ≤ 0.05, **p ≤ 0.01, ****p ≤ 0.0001.

Having determined the neutrophil origin of circulating chromatin after burns, we tested the changes in cNETs over time after 30% total body surface area (TBSA) burn (Fig. 4a). We found a significant surge in cNET concentration immediately after the burn injury (post burn day 1). The cNET values were significantly elevated post-burn compared to the levels before burn. The initial surge was followed by a slower, continuous decrease in the concentration of cNETs during the following week (Figs 4b–d and 5A).

The cNET levels in rats were proportional to the size of burn injury (Fig. 5A). Smaller, comb-burn injuries (5% TBSA) at day 1 post-burn resulted in cNET levels that were comparable to those in control animals. Larger buns (30% TBSA) resulted in significantly higher levels of cNETs.

In a model of double injury (burn and cecal ligation puncture - CLP) the concentration of cNETs increases as soon as 5 hours after the CLP procedure (Fig. 5B). The change in the concentration of cNETs over time follows the same course as the inflammation in the body after a major burn injury (Fig. 5C). While an initial surge after the burn injury is followed by a gradual reduction in the subsequent days, this decrease is reversed following the CLP. The cNETs measured levels increase at 1 day post-CLP and the increase reaches statistical significance at day 2 post-CLP.

## Discussion

We designed a microfluidic device to quantify cNETs in blood. A key feature of the device are the posts arranged in a regular array with precise inter-post distance. The post-array traps chromatin mechanically, by a mechanism analogous to the trapping of hair fibers in a comb. Most chromatin fibers are several hundreds of microns in length. Their length is significantly longer than the space between posts. When these chromatin fibers travel through the array, their ends may enter distinct spaces between posts. The ends travel with the flow, stretching the fiber across the posts, and trapping it in the array. Shorter fibers and oligonucleotides that enter the same space between posts with both ends are not captured and are washed away. The optimal distance between posts for trapping cNETs has been determined based on previous measurements^21^. The amount of chromatin trapped in the first array is proportional to the concentration and volume passed through the devices, as previously shown^21^. The volume of sample and the blood dilution are optimized such that the assay operates within its linear range. A second array is implemented to indicate when the first array has reached its capture capacity limit and chromatin fibers are by-passing it.

The direct comparison between the microfluidic and standard biochemical assays revealed that the microfluidic assay differentiates between chromatin and oligonucleotides whereas the biochemical assay does not. Our method for quantifying intact cNETs directly in blood also avoids the complexity of other methods that are labor intensive and prone to artifacts. Flow cytometry and confocal microscopy methods have high specificity for NETs but they require complex procedures for sample preparation and data analysis^22–24^. These methods also require the removal of red blood cells from the blood sample and involve osmotic changes that could trigger the release of NETs^25^. Our methodology to quantify cNETs circumvents these limitations.

The differences between blood measurements using the microfluidic and PicoGreen assays are intriguing. When applied to blood samples before and after DNase treatment, the differences in the changes recorded by each method reflect the presence of significant amounts of oligonucleotides in the blood sample. When using the PicoGreen assay, the presence of oligonucleotides in blood will result in small changes after the addition of DNase. Inside the microfluidic assay, which is sensitive only to chromatin but not to oligonucleotides, the changes will be a more accurate reflection of the chromatin degradation. Similarly, the addition of PMA to blood samples and the release of cNETs will result in larger changes in the microfluidic assay compared to the PicoGreen assay, in particular when significant amounts of oligonucleotides are already present in the sample. Further studies will determine the correspondence between PicoGreen and microfluidic measurements and enable direct, quantitative comparisons between the two methods.

We validated the neutrophil origin for the trapped chromatin (cNETs) using immunohistochemistry. Moreover, we measured extremely low circulating chromatin levels in neutropenic rats, suggesting that only neutrophils and no other cells damaged by the burn injury are the source of trapped chromatin. Quantitative measurements using the microfluidic assay revealed a rapid surge in cNET concentration immediately after the burn injury, followed by a gradual decrease over the ensuing week. In the days following the cecal ligation puncture, we measured additional increases in the concentration of cNETs, consistent with the robust neutrophil activation during sepsis^26,27^.

Several studies identified the presence of cNETs in the capillaries of the lung during acute respiratory distress and the capillaries of the kidney during acute kidney injury^18,28^. Two mechanisms for the contribution of cNETs to tissue damage have been proposed. A biochemical mechanism is centered on platelet aggregation and fibrin clot formation around chromatin fibers^29,30^. Support for this mechanism is provided by histological observations in neutrophilic, DNase deficient mice^20^. A mechanical mechanism has also been proposed, supported by *in vitro* evidence that NETs can perturb and decouple the traffic of red blood cells and the blood flow in networks of capillaries. In tissues, the perturbed red blood cell distribution in capillary plexuses may result in localized hypoxia and be responsible for secondary organ injury^21^. Support for this hypothesis is provided by observations of increased blood flow in the skin capillary network surrounding small burns after the administration of DNase^28^. Only the mechanical mechanism is dependent of the presence of intact chromatin fibers, while the biochemical mechanism is independent from chromatin length. Thus, methods that can differentiate between chromatin fibers and oligonucleotides in circulation are critically important for studying the mechanisms responsible for complications after burn injuries.

In summary, our study reports on an assay for measuring intact cNETs in blood and differentiating intact cNETs from the oligonucleotides resulting after their degradation. The study was performed in rats and requires further validation with blood samples from patients. While human neutrophils display a lower NETosis threshold compared to rat neutrophils, it is likely that the changes occurring during disease in patients will be detectable using the microfluidic assay. This assay may also be useful for monitoring various other conditions besides burn injuries that involve systemic inflammation.

## Methods

### Rat models of burn injury

All animal experiments were executed in agreement with the guidelines and with permission of the Institutional Animal Care and Use Committee of Massachusetts General Hospital. All procedures were conducted using appropriate anesthesia, analgesia, and aseptic techniques. Wistar male rats, 250–350 g weight (Charles River Laboratories, Wilmington, MA), were employed after acclimatization for 1 week prior to the experiments. During the entirety of the study, the rats were individually housed with free access to food and water.

**Figure 6:**
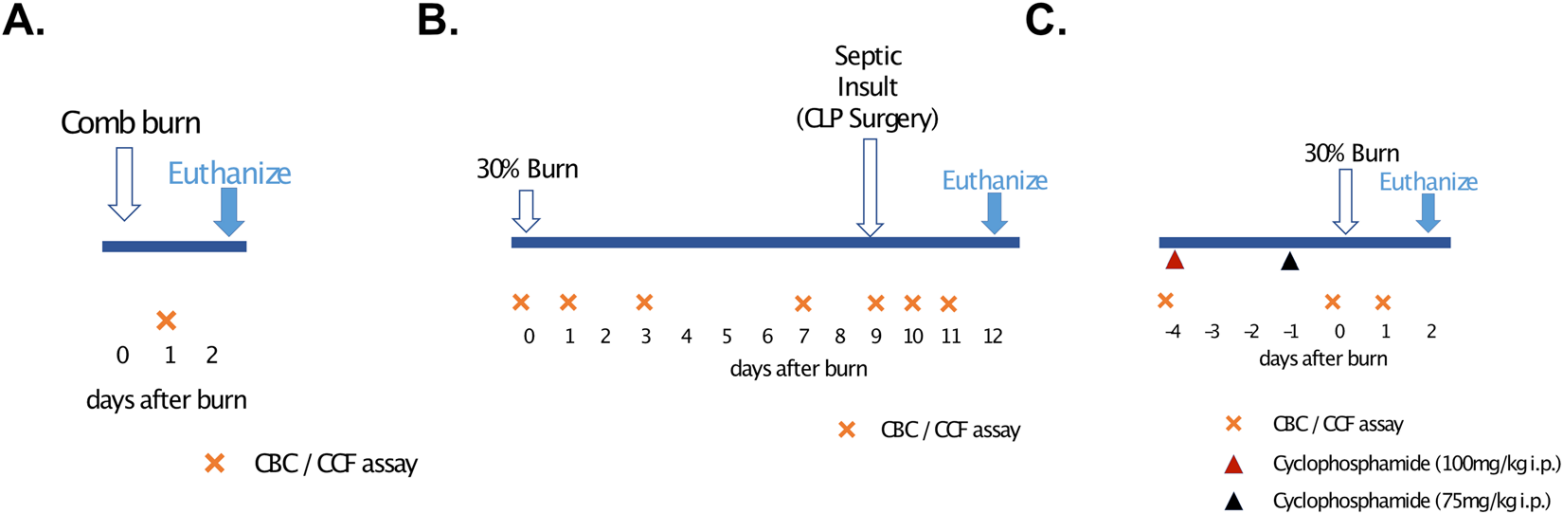
Timeline of the rat experimental procedures. (**A**) In the comb-burn model, blood samples were drawn at 1 post-burn day (PBD). (**B**) In the 30% burn injury, blood samples were drawn at days 1, 3, 9, 11 and 12 PBD. Cecal Ligation Puncture surgery (CLP) was performed at 9 PBD and Blood samples were drawn at 6 hours, 2 and 3 days after CLP (days 9, 11, 12 PBD). One reference blood sample was collected before the burn procedure (0 PBD). (**C**) For the neutropenia model, 100 mg/kg and 75 mg/kg cyclophosphamide were administrated intraperitoneally at 4 and 1 days before the 30% TBSA burn injury. Blood samples were drawn the day before cyclophosphamide injection, right before the burn injury, and 1 PBD.

The procedure for comb burn was performed as previously described, with minor changes^28^. We shaved the animal’s dorsal hair and with a depilatory cream (Magic Razor-less Extra Strength Shave Cream for Men, Soft-sheen Carson) once, just before burn injury. We used a 4-pronged rectangular brass comb to make 2 sets of burn wounds, 1 on each side of the back (1 cm lateral to the dorsal spine). The brass comb had 4 pins, 10 mm wide and 20 mm long, and separated by 5 mm spaces. We immersed the brass comb in boiling water for at least 10 minutes before the injury and applied the hot comb to the back of the animals for 20 seconds. We controlled the force pushing the comb to an estimated 1 N force, the equivalent of 100 g weight pressing the comb against the skin, and this procedure resulted in full-thickness burns. Blood sample was taken at 1PBD and animals were euthanized at 2 PBD (Fig. 6A). We calculated TBSA using the Meeh’s formula (TBSA = *k*W^2/3^, where *k = *10 for laboratory rats and W is the weight of the animal), as previously described^31^. The fraction of TBSA for comb burns was estimated to 3 to 4%.

The procedure for 30% TBSA thermal injury was performed as previously described^18^. The fur of the dorsal surface was shaved and rats were placed in a mold designed to expose precisely 30% of total body surface area to burn injury. The exposed area was immersed in boiling water for 12 seconds to produce a full-thickness burn. Blood samples were taken at 0 (before burn), 1, 7, 9 (6 hours after CLP), 10 and 11 PBD (Fig. 6B). All rats received intraperitoneal fluid resuscitation with 40 mL/kg of normal saline and were kept on a thermostat water blanket until recovered from anesthesia. Buprenorphine at a dose of 0.05 mg/kg was injected subcutaneously before the anesthesia and continued every 12 hours in the first 48 hours after the procedure.

### Secondary CLP surgery

CLP surgery was performed at 9 PBD as previously described^32^, with changes in ligated cecum puncture protocol to induce sepsis after burn. The rats’ abdominal region was shaved and the abdomen was opened with a 2 cm midline incision. The cecum was ligated 1.5 cm distal to the ileocecal valve so that bowel continuity was preserved, and the ligated cecum was punctured once with 21 G needle from mesenteric to anti-mesenteric side. The midline incision was then closed. After surgery, the rats were allowed to recover in their cages with free access to food and water. These rats were surveyed until blood collection at 11 PBD, and all of them were euthanized at 12 PBD (Fig. 6B).

### Neutropenic rat model

Methods for generating a neutropenic rat model were previously described^33^. Briefly, cyclophosphamide was administered intraperitoneally in doses of 100 mg/kg of body weight at 4 days before burn (−4 PBD) and 75 mg/kg of body weight at 1 day before burn (−1 PBD). A 30% TBSA thermal burn was performed at day 0 (0 PBD). Granulocyte counts were taken for non-infected rats to assess the effects of cyclophosphamide at 4 days and just before burn, and at 1 PBD. All rats were monitored closely for 24 hours, and blood samples were collected at 1 PBD and they were euthanized at 2 PBD (Fig. 6C). As demonstrated previously^34^, cyclophosphamide therapy effected a reduction in the mean WBC count from 12,700 WBCs per µL on day 0 to 470 WBCs at day 5 and maintenance of the count at the lower level throughout the therapeutic period (day 5 to 8). Reduction in total WBC count was accompanied by a reduction in granulocytes. On day 0, the mean granulocyte count was 1,905 granulocytes per µL, and on day 5 through 8, it was less than 50 granulocytes per µL. No special precautions were taken with the rats during the period of neutropenia, with no signs of infection and no deaths observed in neutropenic rats.

### Blood sampling and measurement

Venous blood was collected from the saphenous or femoral vein into lithium heparin-coated, 125 µL capillary blood-collection tubes (Safe-t-fill, RAM Scientific, Yonkers, NY). The blood samples were stored at room temperature. We used 25 µL blood for the chromatin microfluidic assay, 50 µL for complete blood counts (CBCs), and discarded the rest. The assays for chromatin and CBCs were performed within 1 hour after blood collection. CBCs were analyzed at the Center for Comparative Medicine veterinary clinical pathology laboratory of Massachusetts General Hospital, using a HemaTrue Hematology Analyzer. This method can analyze three part WBC differential; granulocytes, lymphocytes, and monocytes. Blood films, packed cells volumes, and reticulocyte counts were also manually analyzed whenever clinical abnormalities in the sample were detected by the Hematology Analyzer. Plasma was separated by centrifugation (1500 g for 10 mins in 4 °C) and plasma aliquots were immediately frozen at −80 °C in a freezer.

### Quantification of cf-DNA in plasma

Plasma was separated from blood by centrifugation and diluted 10 times in phosphate buffered saline (1X PBS pH 7.4, Life technologies). The cf-DNA in diluted plasma was quantified using Quant-iT PicoGreen dsDNA kit (Invitrogen Molecular Probes Inc., Eugene, OR, USA) following manufacturer’s instructions. Fluorescence was measured using Synergy 2 plate reader (BioTek Instruments, Inc., Winooski, VT, USA).

### Quantification of cNETs in blood using the microfluidic assay

We designed a microfluidic device that presents a set of posts in offset array (Fig. 1A). The devices were fabricated using standard microfabrication technologies. One layer (35 µm thick) of SU8 photoresist (Microchem, Newton, MA, USA) was patterned on a silicon wafer using photolithography masks and following standard processing recommendations from the manufacturer. The wafer with patterned photoresist was used as a mold to produce pieces of polydimethylsiloxane (PDMS, Fisher Scientific, Fair Lawn, NJ, USA), which were subsequently bonded irreversibly to standard glass slides (1 × 3 inches, Fisher). Up to four devices for CCF assay were bonded together on one glass slide. The devices were fabricated in batches and stored dry at room temperature.

When needed, microfluidic devices were primed with phosphate buffered saline (1X PBS pH 7.4, Life technologies). Blood samples (25 µL), within 1 hour after collection, were diluted in 2 ml of PBS (1:80) and then mixed with 50 µL of 1 mM Sytox-orange (Life technologies) in PBS. The diluted blood was loaded a 1 ml syringe connected to inlet of the microfluidic device. A volume of 200 µL diluted blood was injected into the device using a syringe pump (10 µL/min flow rate, for 20 minutes (Fig. 1B,C). The Sytox-orange Fluorescence was recorded using a Zeiss Axiovert 200 M fluorescence microscope (Carl Zeiss, Oberkochen, Germany) using a Texas Red dye cube (615 nm excitation, 589 nm emission) at room temperature (25 °C). The areas of chromatin trapped in the device was quantified after automatic thresholding using Image J2 (triangle threshold filtration, U.S. National Institutes of Health, Bethesda, MD, USA).

### Immunofluorescence staining of NETs in the microfluidic device

We verified the presence of neutrophil-specific enzymes on chromatin by immunofluorescence staining. First, the device and captured chromatin fibers were blocked by flowing 5% goat serum through the device at 1 µL/min for 2 hours. After blocking, NETs were stained by flowing rabbit anti-neutrophil elastase antibody (1:250 dilution, Abcam ab68672) at 1 µL/min for 30 min, followed by flowing secondary goat anti-rabbit antibodies (1:500, Thermofisher Scientific) at 1 µL/min for 15 min. Finally, nuclear DNA was stained by flowing Sytox-blue (1:2000, Thermofisher Scientific) at 5 µL/min for 15 min.

### NETs-release following PMA stimulation in blood and NETs degradation by DNase

For some experiments, to stimulate the release of NETs from blood neutrophils, 1 µM of PMA was added to a 100 µL blood sample, followed by incubation at 37 °C for 3 hours. To degrade NETs, 10 µL of 10X Turbo DNase buffer and 40 U of turbo DNase were added to 100 µL blood samples pre-incubated with 1 µM PMA for 2 hours at 37 °C. Similar to the blood preparation for the microfluidic assay, 25 µL of PMA stimulated blood was diluted with 2 mL of PBS and 50 µL of 1 mM Sytox-Orange were added to the sample. Subsequently, 3 µL of 10X turbo DNase buffer and 10 U of turbo DNase were added to the samples and incubated at 37 °C for 1 hour.

### Statistical Analysis

Experimental data were depicted as mean ± standard error of the mean (SEM). The number of animals in each experiment and number of experimental repeats are included in figure captions. For the assessment of statistical significance between two sets of data the Mann – Whitney U test was used. For multiple comparisons between more than two sets of data used the one-way analysis of variance (ANOVA). Statistical software used was Prism 7 (version 7.0b, GraphPad Software, La Kolla, CA). Differences were considered to be statistically significant at p ≤ 0.05 (in figures *p ≤ 0.05, **p ≤ 0.01, ***p ≤ 0.001, ****p ≤ 0.0001).
